# New Methods of Esterification of Nanodiamonds in Fighting Breast Cancer—A Density Functional Theory Approach

**DOI:** 10.3390/molecules22101740

**Published:** 2017-10-19

**Authors:** Linda-Lucila Landeros-Martinez, Daniel Glossman-Mitnik, Erasmo Orrantia-Borunda, Norma Flores-Holguín

**Affiliations:** Laboratorio Virtual Nanocosmos, Departamento de Medio Ambiente y Energia, Centro de Investigacion en Materiales Avanzados, Miguel de Cervantes 120, Complejo Industrial Chihuahua, Chihuahua, Chih 31136, Mexico; linda.landeros@cimav.edu.mx (L.-L.L.-M.); daniel.glossman@cimav.edu.mx (D.G.-M.); erasmo.orrantia@cimav.edu.mx (E.O.-B.)

**Keywords:** nanodiamonds, esterification, molecular polar surface area, hydrogen bond, Density Functional Theory

## Abstract

The use of nanodiamonds as anticancer drug delivery vehicles has received much attention in recent years. In this theoretical paper, we propose using different esterification methods for nanodiamonds. The monomers proposed are 2-hydroxypropanal, polyethylene glycol, and polyglicolic acid. Specifically, the hydrogen bonds, infrared (IR) spectra, molecular polar surface area, and reactivity parameters are analyzed. The monomers proposed for use in esterification follow Lipinski’s rule of five, meaning permeability is good, they have good permeation, and their bioactivity is high. The results show that the complex formed between tamoxifen and nanodiamond esterified with polyglicolic acid presents the greatest number of hydrogen bonds and a good amount of molecular polar surface area. Calculations concerning the esterified nanodiamond and reactivity parameters were performed using Density Functional Theory with the M06 functional and the basis set 6–31G (d); for the esterified nanodiamond–Tamoxifen complexes, the semi-empirical method PM6 was used. The solvent effect has been taken into account by using implicit modelling and the conductor-like polarizable continuum model.

## 1. Introduction

Breast cancer is a leading cause of death worldwide. This disease is mainly characterized by high levels of estrogen receptor (ER) and progesterone receptor (PR) expressions [1]. One of the treatments prescribed for patients with breast cancer is tamoxifen (TAM) [2], which belongs to the family of selective estrogen receptor modulators (SERMs) [3]. Nonetheless, prolonged use of TAM causes adverse effects such as endometrial cancer, thromboembolisms, and menopause symptoms [4,5].

Fortunately, nanomedicine has demonstrated exceptional promise for improving the delivery of therapeutic agents for the treatment of breast cancer through nanomaterials and, in particular, with nanodiamonds (ND). These nanomolecules are being studied due to their unique characteristics that make them suitable as a platform for diagnosis and therapeutics [6]. In addition, ND are stable and dispersible in water, making them a promising and clinically important modality for improving the efficacy of the treatment of diseases, and even some cancers, at the molecular level [7]. This carbon-based material is medically relevant because of its biological applications and its physical and chemical properties including stability, scalability, small size, good adsorption, and biocompatibility [6,8,9]. ND are known for easily forming complexes with biopolymers [10] and for their many uses [11]. All these properties allow for the modification of its surface with functional groups through covalent and non-covalent bonds and through the attachment of medically relevant agents onto the particle [6]. In practice, ND have been used with a range of polymers such as polyethylene glycol (PEG), polylactide-co-glycolide, and polysaccharide- and amino acid-based polymers. These uses have been successfully implemented, particularly for delivering bioactives through the circulatory system [12]. No use of ND has been reported with 2-hydroxypropanal (2HP) because this monomer has been used as a biocatalyst for the stereoselective production of a compound that allows it to have therapeutic action [13]. Meanwhile, polyglicolic acid (PGA) has been used to yield a variety of different structures, including fibers, follow tubes, porous sponges [14,15], and non-woven meshes, which are materials used in cell delivery [16].

The functional groups on the surface of ND can also interact electrostatically or chemically with other molecules in a sample under study for cell-specific interaction and targeting [17]. This electrostatic interaction or hydrogen bonds (HBs) between the drug and a carboxyl or hydroxyl group of functionalized ND [18,19,20,21] are studied in this research. Theoretical studies on the chemical properties of the ND-TAM complex are important for gaining a better understanding of the interaction between this complex and hormonal receptors. Several studies have investigated using the Density Functional Theory (DFT) to calculate properties of carbon-based nanostructured materials with excellent approximations when compared to experimental data. Gueorguiev et al. compared the energy cost for the formation of dangling bonds in fullerene-like Carbon Nitride (CNx), Phosphorus-Carbide (CPx), and amorphous Carbide (a-C) [22]; probed the energy cost obtained by the Synthetic Growth Concept (SGC) based on the DFT on P atoms inserted in fullerene-like Phosphorus Carbide (FL-CPx) [23]; and made a comparison of the DFT predictions of the infrared (IR) spectra of clusters of Ti_8_C_12_ Zr_8_C_12_ [24].

Additionally, recent studies developed the M06 functional and 6–31G(d) basis sets to load tamoxifen onto the surface of ND by molecular modeling techniques [25]. Some theoretical studies have been reported for polymers, including the mechanism of the addition of organomagnesium reagents to 2HP through an ab-initio analysis [26], the addition of organomagnesium compounds to 2HP with the semi-empirical method PM3 and HF/3–21G to identify the stationary points on the potential energy surface [27], and comparative studies of the differences between the normal modes of polyethylene and polyethylene glycol with the B3LYP and Hartree–Fock methods with several basis sets [28]. Kimani et al. [29] carried out a theoretical analysis of mono-, di-, and tri-PEG conjugated with chlorine for the removal of ovarian cancer cells. Finally, a comparison of the theoretical energy of PGA has been calculated with MP2, B3LYP, and BLYP techniques with the 6–31G(d) basis set [30].

The main objective of this study is to investigate new methods for esterification in nanodiamonds through molecular modeling techniques, using 2-hydroxypropanal, polyethylene glycol, and polyglicolic acid. The esterification facilitates the loading of tamoxifen onto the nanodiamond by means of electrostatic interactions, particularly hydrogen bonds (HB). These HBs are formed between the linear chain generated with the esterification and the linear chain of TAM, composed of an ether and a tertiary amine. This coupling allows the drug to be delivered specifically to the hormone receptors, thus improving its efficacy.

## 2. Materials and Methods

The computational studies of the construction of esterified nanodiamond (NDE), esterified nanodiamond complexes (NDE-TAM), and electronic properties were performed using Gaussian 09 software [31]. Some of the systems were developed using the Density Functional Theory (DFT) method [32,33] while the systems with a huge number of atoms were optimized with semi-empirical methods. The details are in the next subsections:

### 2.1 Esterified Nanodiamond (NDE)

Analysis and characterization of the esterified nanodiamond (NDE) was calculated with the hybrid meta-generalized gradient approximation (meta-GGA) functional M06 [34,35] combined with the basis set 6–31G(d) [36], and the conductor-like polarizable continuum model [37] was used with water as solvent.

### 2.2. Esterified Nanodiamond Complexes (NDE-TAM)

The optimization of the esterified nanodiamond complex (NDE-TAM) was performed using the semi-empirical method PM6 [38] (to achieve convergence of the multi-atom complex), taking into account the good past results on the structural parameters of the method [39,40,41]. Water was used as the solvent with the conductor-like polarizable continuum model.

### 2.3. Reactivity Parameters

The electronic properties of NDE and the NDE-TAM complex were calculated by means of M06/6–31G(d). The solvation model was a conductor-like polarizable continuum model, with water as the solvent. The DFT approach has been successfully used for chemical descriptors such as electron affinity (EA), ionization potential (IP), chemical hardness (η) [42], electronegativity (χ) [43,44], chemical potential (μ) [45], and electrophilicity (ω) [46]. These reactivity descriptors were obtained by single point calculations, considering the energy (E) as a function of the number of electrons, N: E(N − 1), E(N), and E(N + 1). Approximations were also done of IP and AE to HOMO and LUMO energies obtained from the Kohn–Sham scheme.

### 2.4. Molecular Polar Surface Area

The molecular polar surface area (PSA) was obtained using a commercial package based on Bayesian statistics (Molinspiration miscreen toolkit) [47]. To obtain the PSA, the NDE and the NDE-TAM complex were encoded with SMILE (Simplified Molecular Input Line System), which is a chemical notation system designed for modem chemical information processing [48].

## 3. Results and Discussion

### 3.1. Construction of the Proposed Systems

Nanodiamond is often described as a crystalline diamond core with a perfect diamond lattice surrounded by an amorphous shell with a combination of sp2 and sp3 bonds [49]. The constructed ND for this work is octahedral with 351 atoms with 111 facets and a lattice constant of 3.56 Å. The size of the nanodiamond used is 1.5 nm due to the stability of the NDs in the range between 1.5 and 2.0 nm [50,51].

The functionalization of the ND begins with the creation of carboxyl functional groups on its surface, simulating acid treatment [52,53]. Three different molecules were used to create the esterification: 2-hydroxypropanal (2HP), polyethylene glycol (PEG), and polyglicolic acid (PGA) monomers. The construction of the esterified structures was carried out following the reaction protocol established by Landeros-Martinez et al. [25]. This process involves bonding the carboxyl group on the surface of the nanodiamond in a covalent bond with the different monomers proposed in this work. This generates an available extended linear chain on the surface of the nanodiamond.

Also, the analysis of nucleophilicity index of the alcohols and the electrophilicity index of the ND was taken into account in the reactivity section. The simplest approach was used: considering that high (low) nucleophilicities are associated with low (high) ionization potentials, nucleophilicity was related with a negative IP value. The nucleophilicity index was calculated in the manner introduced by Domingo et al. [54]. Tetracyanoethylene (TCE) is taken as a reference for this scale of nucleophilicity because it presents the lowest HOMO energy in a large series of molecules already considered.

*N*_(Nu)_ = E_HOMO(Nu)_ (eV) − E_HOMO(TCE)_ (eV) (1)

E_HOMO(TCE)_ = −9.13 eV calculated with the methodology mentioned in the computational details section (M06/6–31G(d)).

The alcohols showed low nucleophilicity index values and high values of electrophilicity, characterizing them as marginal nucleophiles and strong electrophiles according to Domingo et al. [55]. However, the ND has a strong nucleophile character with *N* > 3.0 eV. Results are in Table 1.

**Figure 1:**
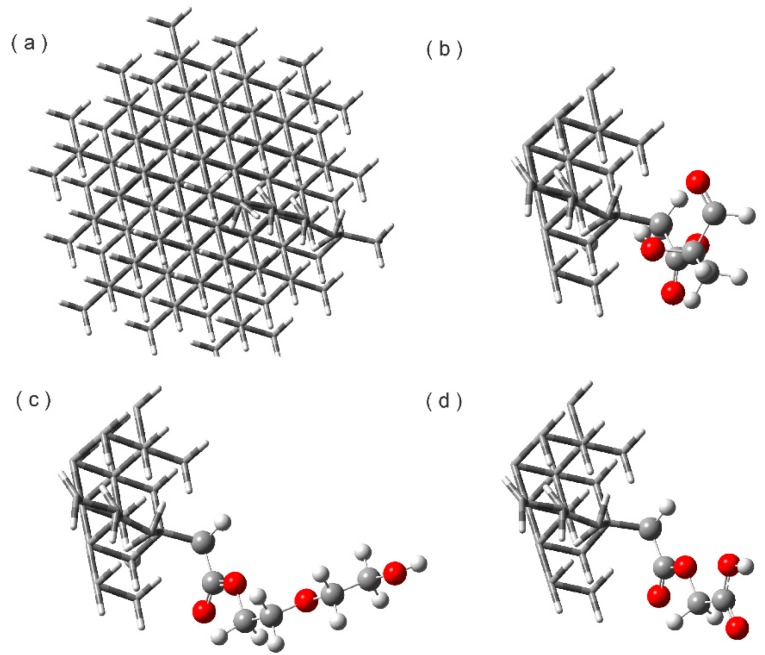
Optimized structures: (**a**) Nanodiamond; (**b**) ND2HP; (**c**) NDPEG; and (**d**) NDPGA at the M06/6–31G(d) level of theory.

**Figure 2:**
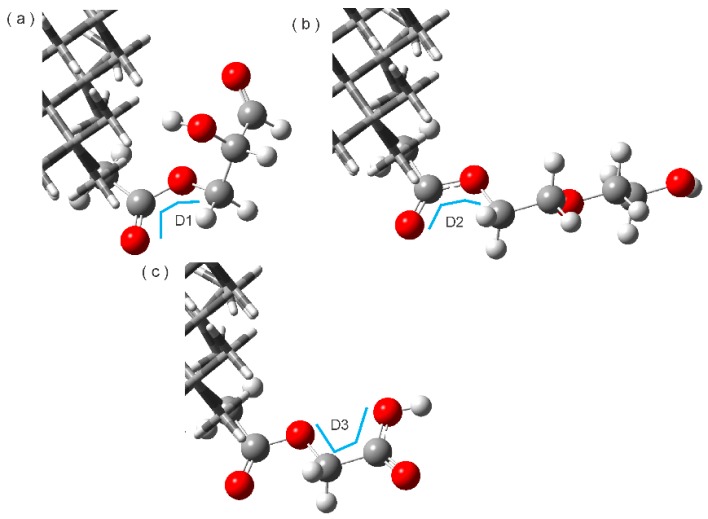
Dihedral D1, D2, and D3 in (**a**) ND2HP; (**b**) NDPEG; and (**c**) NDPGA.

After the construction of the systems, they were optimized in aqueous phase and named ND2HP, NDPEG, and NDPGA. Once the optimization was reached, frequency calculations were performed to ensure that the structures were at their lowest energy level. The structures are shown in Figure 1 where the view is from the top of the ND. We observed that the structure of the ND did not change before or after optimization. However, the linear chain of esterification had significant changes in its dihedral angles. These changes will outline the position of the TAM drug for its loading in the nanoparticle. After optimization in 2HP the D1 (O352–C351–O353–C354) changed from 3.314 to −14.42 degrees. PEG with dihedral D2 (O352–C351–O353–C354) changed from 3.314 to −1.88. The dihedral D3 (O353–C354–C355–O356) in PGA changed from −118.47 to −1.57 degrees. The dihedrals D1, D2, and D3 are in Figure 2 and identified with a blue line.

### 3.2. Reactivity Parameters of the NDE

The following reactivity parameters were obtained: electron affinity (EA), ionization potential (IP), electronegativity (χ), chemical potential (μ), chemical hardness (η), and electrophilicity (ω). The reactivity parameters mentioned above were obtained using two different approximations. As stated in the computational details section, the first considered the energy (E) as a function of the number of electrons, and the second one used the approximation of IP and EA to HOMO and LUMO energies. Results are in Table 2, where the second approximation values are in cursive font.

The values in Table 2 suggest greater reactivity for ND2HP. It has the lowest hardness in both approximations, and has the highest value for electrophilicity index even when the three of the esterified systems are classified as moderate electrophiles, in accordance with Domingo et al. [55] with ω < 1.5 eV. In this work, the main area of interest is that the reaction with TAM and ND2HP showed the highest tendency to attract electrons and presented better stability with the electrons that were in the surrounding area.

Another important feature that quantifies permeation is polar surface area (PSA). This property is defined as the surface sum of all the polar atoms, including the oxygen, nitrogen, and hydrogen attached to these atoms [56]. PSA is a useful parameter for the prediction of drug transport properties. Molecules with value greater than 140 Å^2^ are poorly absorbed at cell membranes, while those with a value less than 90 Å^2^ are completely absorbed [57]. The results were 63.60 Å^2^ for ND2HP and NDPGA, and 55.70 Å^2^ for NDPEG, meaning that all the proposed systems have good cell permeability.

### 3.3. Construction of the NDE-TAM Complex

**Figure 3:**
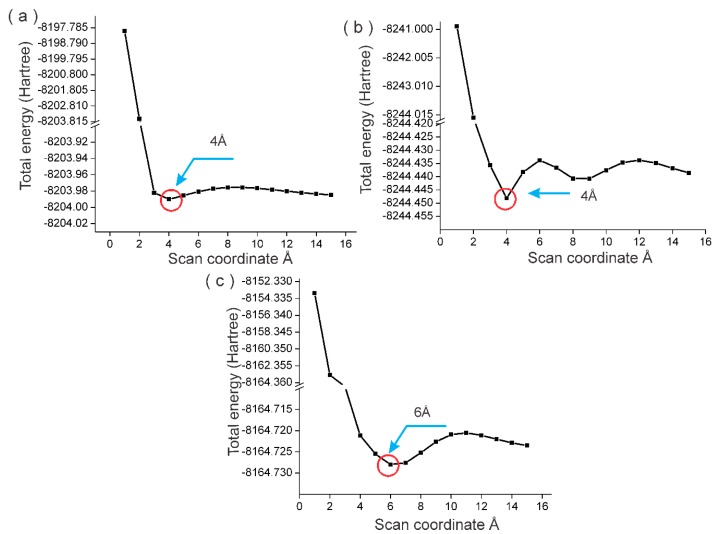
Energy curve of binding distance for (**a**) ND2HP and tamoxifen (TAM); (**b**) NPEG and TAM; and (**c**) NDPGA and TAM.

To build the NDE-TAM complex, it was necessary to find the equilibrium distance between the NDE and the drug. With the optimized structure obtained in the previous section, an equilibrium distance analysis was performed. This exploration involved searching for the distance requiring the least energy to bind a pair of molecules—in this case, NDE and TAM. The analysis consisted of single-point energy calculations across a rectangular grid involving a scan of previously selected internal coordinates. When a scan was requested, the variables in the molecular structure, and the range of values which they could take, had to be specified. These variables were defined according to the structure systems and the intended bond type. The scan was performed in 15 steps with approximations of 1 Å. The atoms involved in the approximation are a hydrogen of the NDE, an oxygen in TAM in ND2HP and NDPEG, and two oxygens in NDPGA. The rest of the atoms remained fixed. The energy curve results were 4 Å for ND2HP-TAM and NDPEG-TAM, and 6 Å for NDPGA-TAM. The three different curves obtained are shown in Figure 3.

Considering that the scan helps to determine the approximate location of the minimum energy structure, but does not include optimized geometry [58], all the NDE-TAM complexes were optimized. In this case, the geometry optimization and the frequency calculation were developed using the PM6 semi-empirical method, using water as solvent due to the size of the system. The results on the binding bonds are 7.79 Å for ND2HP-TAM, 4.21 Å for NDPEG-TAM, and 2.18 Å for NDPGA-TAM.

### 3.4. Hydrogen Bonds

**Figure 4:**
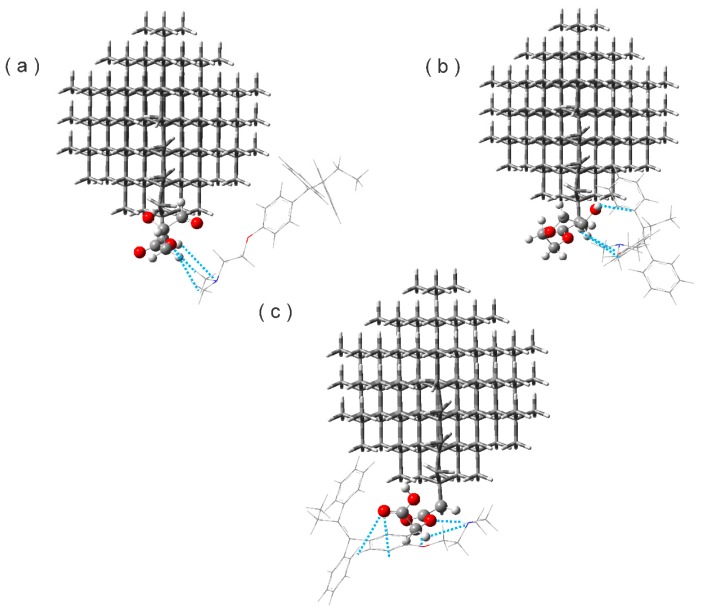
Hydrogen bonding in the (**a**) ND2HP-TAM complex; (**b**) NDPEG-TAM complex; and (**c**) NDPGA-TAM complex.

The hydrogen bond is also one of the most important intermolecular interactions because it determines molecular conformation, molecular aggregation, and the function of a vast number of chemical systems ranging from inorganic to biological [59]. Based on the optimized structures of the NDE-TAM complexes, the counting analysis led to the definition of different HBs such as C=O---H-C, H-O----C, C-H----N, and C-H----O. See Figure 4.

The distance and angles observed in the HBs are classified as weak, according to Jeffrey classification [60]. The results in Table 3 show the type of HBs and the interaction type, where the groups C–H and O–H act as proton donors. These kinds of interactions are possible, even when the groups have slight polarity, as stated by Steiner [59]. N and O act as acceptors.

**Figure 5:**
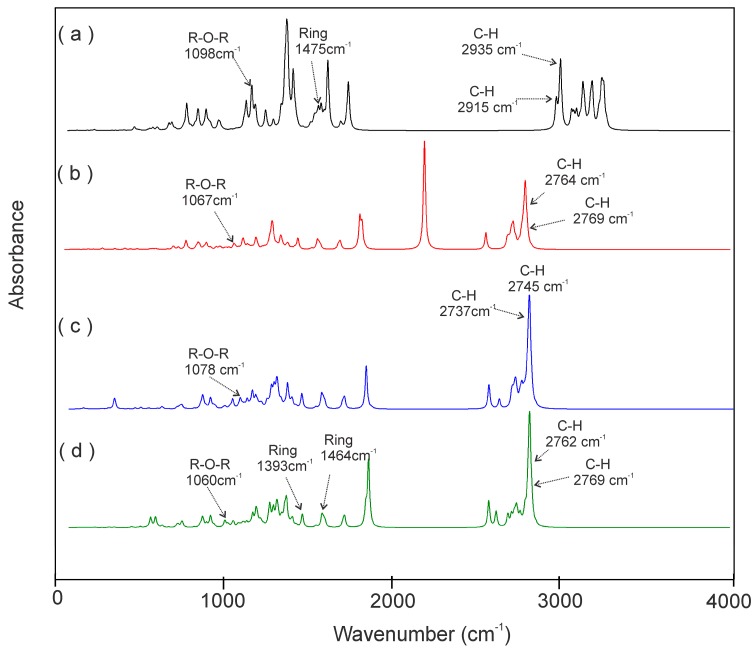
Infrared (IR) spectra of (**a**) TAM; (**b**) ND2HP-TAM complex (PM6); (**c**) NDPEG-TAM complex; and (**d**) NDPGA-TAM complex.

The presence of the hydrogen bonds found was corroborated qualitatively with an analysis of the IR spectra of the free A–H group with no HB group. According to Desiraju, in a comparison of the IR spectrum of a free A–H group and another one with a HB, in the HB, the A–H stretching frequency is reduced. This indicates a hydrogen bonding [61]. For our study, we compare the TAM’s interaction with the A-H----B hydrogen bond group in the NDE-TAM complexes. The bonding of the atoms to H decreases the vibration frequency. Figure 5 shows the IR spectrum of TAM at the M06/6–31G(d) level [25] versus the IR spectra of the ND2HP-TAM, NDPEG-TAM, and NDPGA-TAM complexes. The latter three use the PM6 semi-empirical method. The comparison shows the energy reduction on the frequencies as follows: The C–H stretching frequency of 2935 cm^−1^ in TAM decreases to 2769, 2745, and 2769 cm^−1^ for ND2HP-TAM, NDPEG-TAM, and NDPGA-TAM, respectively. C–H stretching at 2915 cm^−1^ in TAM decreases to 2764 cm^−1^ in ND2HP-TAM, 2737 cm^−1^ in NDPEG-TAM, and 2762 cm^−1^ in NDPGA-TAM.

The ring vibration is present at 1475 cm^−1^ in TAM, and at 1393 cm^−1^ and 1464 cm^−1^ in the NDPGA-TAM complex. The R-O-R vibration in TAM is 1098 cm^−1^, while in the ND2HP-TAM, NDPEG-TAM, and NDPGA-TAM complexes the R-O-R vibration is 1067 cm^−1^, 1078 cm^−1^, and 1060 cm^−1^, respectively.

According to Lipinski´s rule of five, which states that poor permeation is more likely when there are more than five HB, the number of these bonds plays a crucial role in determining if a drug will be more active [62]. The three analyzed NDE-TAM complexes fulfill this rule.

The PSA was calculated at 12.47 Å^2^ for the three complexes. This is due to the number of polar oxygen and nitrogen atoms and number of hydrogen atoms attached to the oxygen. The three systems have the same number of these atoms, creating good cell permeability.

### 3.5. Chemical Reactivity Descriptors of NDE-TAM

A quantitative analysis of the reactivity of the NDE-TAM complexes was performed by means of the global reactivity descriptors. The values of the global reactivity descriptors, calculated for each of the NDE-TAM complexes using the vertical electron affinity and ionization potential, are shown in Table 4.

The results of chemical reactivity show similar values for each of the complexes studied. ND2HP-TAM shows the highest value for EA and IP, therefore, the highest electrophilicity index with 1.53/*1.42* eV. The value calculated with the energies approximation places ND2HP-TAM as strong, while the result for the HOMO-LUMO approximation places it as a moderate electrophile, and therefore close to the level of strong electrophiles. The Nucleophilicity indices calculated for these systems are 6.06, 6.14, and 6.16 eV for NDE2HP, NDEPEG, and NDEPGA, respectively. All three are classified as strong nucleophiles.

## 4. Conclusions

The esterification of nanodiamond with 2-hydroxypropanal, polyethylene glycol, and polyglicolic acid allowed the generation of a longer linear chain in the carrier vehicle that interacts electrostatically with the linear chain of the drug tamoxifen. The nanodiamond esterified with 2-hydroxipropanal showed the highest reactivity. The three molecular systems proposed as new esterification models have good permeability in cells according to their PSA. The hydrogen bond analysis showed that NDPGA-TAM generates a greater number of hydrogen bonds. The presence of HBs was corroborated with the vibrational frequencies analysis. The reactivity parameters show ND2HP-TAM as the only strong electrophile. NDPEG-TAM and NDPGA-TAM are classified as moderate electrophiles, in accordance with classification criteria by Domingo et al. The three proposed complexes have good biodistribution, permeability, and pharmacological action on cancer cells, according to Lipinski’s Law.

## Figures and Tables

**Table 1:** Nucleophilicity *N* and electrophilicity ω indexes of esterification reaction.

Molecules	*N* (eV)	ω (eV)
Nanodiamond (ND)	6.18	0.81
2-Hydroxypropanal (2HP)	1.87	2.82
Polyethylene glycol PEG	1.91	4.28
Polyglicolic acid (PGA)	1.78	5.96

**Table 2:** Reactivity parameters of esterified ND calculated with energies approximation/*HOMO-LUMO approximation*.

ND_Esterified_	EA (eV)	IP (eV)	η (eV)	χ = −μ (eV)	ω (eV)
ND2HP	0.53/*0.50*	2.73/*2.99*	1.11/*1.25*	1.64/*1.74*	1.21/*1.22*
NDPEG	−0.25/−*0.35*	2.71/*2.96*	1.48/*1.66*	1.23/*1.30*	0.51/*0.51*
NDPGA	0.07/−*0.11*	2.72/*2.99*	1.32/*1.55*	1.39/*1.44*	0.73/*0.67*

**Table 3:** Hydrogen bonding in NDE-TAM complexes.

Complex	Interaction Type	Bond Lengths A----B (Å)	Bond Angles (A-H----B) (°)	Type
ND2HP-TAM	C-H----O	3.38	164.48	Weak
	C-H----O	3.38	110.75	Weak
	C-H----N	4.69	149.21	Weak
	C-H----O	4.2	124.87	Weak
NDPEG-TAM	C-H----O	4.2	89.35	Weak
	O-H----C	4.01	132.79	Weak
	C-H----O	3.31	158.41	Weak
	C-H----N	3.96	129.22	Weak
NDPGA-TAM	C-H----O	3.12	138.53	Weak
	C-H----O=C	3.57	91.74	Weak
	C-H----O=C	4.07	92.05	Weak

**Table 4:** Reactivity parameters of complexes calculated with energies approximation/*HOMO-LUMO approximation*.

Complex	EA (eV)	IP (eV)	η (eV)	χ = −μ (eV)	ω (eV)
ND2HP-TAM	0.75/*0.64*	2.84/*3.08*	1.05/*1.22*	1.79/*1.86*	1.53/*1.42*
NDPEG-TAM	0.66/*0.35*	2.75/*3.00*	1.05/*1.33*	1.72/*1.67*	1.39/*1.05*
NDPGA-TAM	0.70/*0.41*	2.73/*2.98*	1.02/*1.29*	1.72/*1.69*	1.45/*1.11*

## References

[1] Wang X., Chen X., Yang X., Gao W., He B., Dai W., Zhang H., Wang X., Wang J., Zhang X. (2016). A nanomedicine based combination therapy based on qlpvm peptide functionalized liposomal tamoxifen and doxorubicin against luminal a breast cancer. Nanomed. Nanotechnol. Biol. Med..

[2] Altmeyer C., Karam T.K., Khalil N.M., Mainardes R.M. (2016). Tamoxifen-loaded poly(l-lactide) nanoparticles: Development, characterization and in vitro evaluation of cytotoxicity. Mater. Sci. Eng. C.

[3] Khan M.M., Wakade C., De Sevilla L., Brann D.W. (2015). Selective estrogen receptor modulators (serms) enhance neurogenesis and spine density following focal cerebral ischemia. J. Steroid Biochem. Mol. Biol..

[4] Rossini M., Lello S., Sblendorio I., Viapiana O., Fracassi E., Adami S., Gatti D. (2013). Profile of bazedoxifene/conjugated estrogens for the treatment of estrogen deficiency symptoms and osteoporosis in women at risk of fracture. Drug Des. Dev. Ther..

[5] Lim Y.P., Lin C.L., Lin Y.N., Ma W.C., Hung D.Z., Kao C.H. (2015). Tamoxifen treatment and the reduced risk of hyperlipidemia in asian patients with breast cancer: A population-based cohort study. Clin. Breast Cancer.

[6] Man H.B., Ho D. (2012). Nanodiamonds as platforms for biology and medicine. J. Lab. Autom..

[7] Zhang X.-Q., Chen M., Lam R., Xu X., Osawa E., Ho D. (2009). Polymer-functionalized nanodiamond platforms as vehicles for gene delivery. ACS Nano.

[8] Kaur R., Badea I. (2013). Nanodiamonds as novel nanomaterials for biomedical applications: Drug delivery and imaging systems. Int. J. Nanomed..

[9] Mostofizadeh A., Li Y., Song B., Huang Y. (2011). Synthesis, properties, and applications of low-dimensional carbon-related nanomaterials. J. Nanomater..

[10] Lim D.G., Prim R.E., Kim K.H., Kang E., Park K., Jeong S.H. (2016). Combinatorial nanodiamond in pharmaceutical and biomedical applications. Int. J. Pharm..

[11] Xiao J., Duan X., Yin Q., Zhang Z., Yu H., Li Y. (2013). Nanodiamonds-mediated doxorubicin nuclear delivery to inhibit lung metastasis of breast cancer. Biomaterials.

[12] Khandare J., Calderón M., Dagia N.M., Haag R. (2012). Multifunctional dendritic polymers in nanomedicine: Opportunities and challenges. Chem. Soc. Rev..

[13] Takahashi H., Kobayashi Y., Kaneko N. (1983). Conformational studies of dl-lactaldehyde by 1h-nmr, raman and i.r. Spectroscopy. Spectrochim. Acta Part A Mol. Spectrosc..

[14] Frazza E., Schmitt E. (1971). A new absorbable suture. J. Biomed. Mater. Res..

[15] Mooney D., Organ G., Vacanti J., Langer R. (1993). Design and fabrication of biodegradable polymer devices to engineer tubular tissues. Cell Transplant..

[16] Mooney D.J., Mazzoni C.L., Breuer C., McNamara K., Hern D., Vacanti J.P., Langer R. (1996). Stabilized polyglycolic acid fibre-based tubes for tissue engineering. Biomaterials.

[17] Chung P.H., Perevedentseva E., Tu J.S., Chang C.C., Cheng C.L. (2006). Spectroscopic study of bio-functionalized nanodiamonds. Diam. Relat. Mater..

[18] Ho D., Wang C.-H.K., Chow E.K.-H. (2015). Nanodiamonds: The intersection of nanotechnology, drug development, and personalized medicine. Sci. Adv..

[19] Zhu Y., Li W.X., Li Q.N., Li Y.G., Li Y.F., Zhang X.Y., Huang Q. (2009). Effects of serum proteins on intracellular uptake and cytotoxicity of carbon nanoparticles. Carbon.

[20] Zhu Y., Ran T.C., Li Y.G., Guo J.X., Li W.X. (2006). Dependence of the cytotoxicity of multiwalled carbon nanotubes on the culture medium. Nanotechnology.

[21] Li Y.Q., Tong Y.L., Cao R.X., Tian Z.M., Yang B.S., Yang P. (2014). In vivo enhancement of anticancer therapy using bare or chemotherapeutic drug-bearing nanodiamond particles. Int. J. Nanomed..

[22] Gueorguiev G.K., Broitman E., Furlan A., Stafström S., Hultman L. (2009). Dangling bond energetics in carbon nitride and phosphorus carbide thin films with fullerene-like and amorphous structure. Chem. Phys. Lett..

[23] Gueorguiev G.K., Czigány Z., Furlan A., Stafström S., Hultman L. (2011). Intercalation of p atoms in fullerene-like cpx. Chem. Phys. Lett..

[24] Gueorguiev G., Pacheco J. (2003). Shapes of cagelike metal carbide clusters: First-principles calculations. Phys. Rev. B.

[25] Landeros-Martinez L.-L., Chavez-Flores D., Orrantia-Borunda E., Flores-Holguin N. (2016). Construction of a Nanodiamond Tamoxifen Complex as a Breast Cancer Drug Delivery Vehicle. J. Nanomater..

[26] Oliva M., Safont V.S., Andrés J., Castillo R., Moliner V. (1997). Understanding the mechanism of the addition of organomagnesium reagents to 2-hydroxypropanal: An ab initio molecular orbital analysis. Int. J. Quantum Chem..

[27] Safont V.S., Moliner V., Oliva M., Castillo R., Andrés J., González F., Carda M. (1996). A theoretical study of addition of organomagnesium reagents to chiral α-alkoxy carbonyl compounds. J. Org. Chem..

[28] Talebian E., Talebian M. (2014). A comparative dft study on the differences between normal modes of polyethylene and polyethylene glycol via b3lyp hamiltonian and the hartree-fock method in multiple bases. Optik-Int. J. Light Electron Opt..

[29] Kimani S., Ghosh G., Ghogare A., Rudshteyn B., Bartusik D., Hasan T., Greer A. (2012). Synthesis and characterization of mono-, di-, and tri-poly(ethylene glycol) chlorin e6 conjugates for the photokilling of human ovarian cancer cells. J. Org. Chem..

[30] Blomqvist J., Mannfors B., Pietilä L.O. (2000). Studies on aliphatic polyesters. Part ii. Ab initio, density functional and force field studies of model molecules with two carboxyl groups. J. Mol. Struct. THEOCHEM.

[31] Frisch M.J.T., Trucks G.W., Schlegel H.B., Scuseria G.E., Robb M.A., Cheeseman J.R., Scalmani G., Barone V., Mennucci B., Petersson G.A. (2009). Gaussian 09.

[32] Hohenberg P., Kohn W. (1964). Inhomogeneous electron gas. Phys. Rev..

[33] Kohn W., Sham L.J. (1965). Self-consistent equations including exchange and correlation effects. Phys. Rev..

[34] Zhao Y., Truhlar D.G. (2008). Density functionals with broad applicability in chemistry. Acc. Chem. Res..

[35] Zhao Y., Truhlar D.G. (2008). The M06 suite of density functionals for main group thermochemistry, thermochemical kinetics, noncovalent interactions, excited states, and transition elements: Two new functionals and systematic testing of four m06-class functionals and 12 other functionals. Theor. Chem. Acc..

[36] Rassolov V.A., Ratner M.A., Pople J.A., Redfern P.C., Curtiss L.A. (2001). 6-31G* basis set for third-row atoms. J. Comput. Chem..

[37] Tomasi J., Persico M. (1994). Molecular interactions in solution: An overview of methods based on continuous distributions of the solvent. Chem. Rev..

[38] Stewart J.J.P. (2007). Optimization of parameters for semiempirical methods v: Modification of nddo approximations and application to 70 elements. J. Mol. Model..

[39] Eto M., Yamaguchi K., Shinohara I., Ito F., Yoshitake Y., Harano K. (2011). Role of edge-to-face interaction between aromatic rings in clathrate formation of 1-benzoyl-2-hydroxyindoline derivatives with benzene. X-ray crystal and pm6 analyses of the interaction. Tetrahedron.

[40] Temelso B., Alser K.A., Gauthier A., Palmer A.K., Shields G.C. (2014). Structural analysis of α-fetoprotein (afp)-like peptides with anti-breast-cancer properties. J. Phys. Chem. B.

[41] Benghodbane S., Khatmi D. (2012). A theoretical study on the inclusion complexation of doxycycline with crysmeb. C. R. Chim..

[42] Pearson R.G. Absolute electronegativity and hardness correlated with molecular orbital theory. Proceedings of the National Academy of Sciences of the United States of America.

[43] Putz M.V., Russo N., Sicilia E. (2003). Atomic radii scale and related size properties from density functional electronegativity formulation. J. Phys. Chem. A.

[44] Parr R.G., Donnelly R.A., Levy M., Palke W.E. (1978). Electronegativity: The density functional viewpoint. J. Chem. Phys..

[45] Robert G., Parr Y.W. (1989). Density-Functional Theory of Atoms and Molecules.

[46] Parr R.G., Szentpály L.V., Liu S. (1999). Electrophilicity index. J. Am. Chem. Soc..

[47] Cheminformatics M. Bratislava. Slovak Republic Home Page. http://www.molinspiration.com/services/properties.html.

[48] Weininger D. (1988). SMILES, a chemical language and information system. 1. Introduction to methodology and encoding rules. J. Chem. Inf. Comput. Sci..

[49] Ho D.N. (2010). Applications in Biology and Nanoscale Medicine.

[50] Mochalin V.N., Shenderova O., Ho D., Gogotsi Y. (2012). The properties and applications of nanodiamonds. Nat. Nano.

[51] Stehlik S., Varga M., Ledinsky M., Jirasek V., Artemenko A., Kozak H., Ondic L., Skakalova V., Argentero G., Pennycook T. (2015). Size and purity control of hpht nanodiamonds down to 1 nm. J. Phys. Chem. C.

[52] Pichot V., Bonnot K., Piazzon N., Schaefer M., Comet M., Spitzer D. (2010). Deposition of detonation nanodiamonds by langmuir-blodgett technique. Diam. Relat. Mater..

[53] Marc C., Vincent P., Benny S., Fabienne B., Denis S. (2010). Detonation nanodiamonds for doping kevlar. J. Nanosci. Nanotechnol..

[54] Domingo L.R., Chamorro E., Pérez P. (2008). Understanding the Reactivity of Captodative Ethylenes in Polar Cycloaddition Reactions. A Theoretical Study. J. Org. Chem..

[55] Domingo L.R., Rios-Gutierrez M., Pérez P. (2016). Applications of the Conceptual Density Functional Theory Indices to Organic Chemistry Reactivity. Molecules.

[56] Ertl P. (2008). Polar Surface Area.

[57] Clark D.E. (1999). Rapid calculation of polar molecular surface area and its application to the prediction of transport phenomena. 1. Prediction of intestinal absorption. J. Pharm. Sci..

[58] Frisch J.B.F.a.Æ. (1996). Exploring Chemistry with Electronic Structure Methods.

[59] Steiner T. (2002). The hydrogen bond in the solid state. Angew. Chem. Int. Ed..

[60] Jeffrey G.A. (1997). An Introduction to Hydrogen Bonding.

[61] Desiraju G.R., Steiner T. (2001). The Weak Hydrogen Bond: In structural Chemistry and Biology.

[62] Lipinski C.A., Lombardo F., Dominy B.W., Feeney P.J. (2001). Experimental and computational approaches to estimate solubility and permeability in drug discovery and development settings. Adv. Drug Deliv. Rev..

